# Disabling a Type I-E CRISPR-Cas Nuclease with a Bacteriophage-Encoded Anti-CRISPR Protein

**DOI:** 10.1128/mBio.01751-17

**Published:** 2017-12-12

**Authors:** April Pawluk, Megha Shah, Marios Mejdani, Charles Calmettes, Trevor F. Moraes, Alan R. Davidson, Karen L. Maxwell

**Affiliations:** a Department of Biochemistry, University of Toronto, Toronto, Ontario, Canada; b Department of Molecular Genetics, University of Toronto, Toronto, Ontario, Canada; Max Planck Institute for Infection Biology

**Keywords:** CRISPR-Cas, *Pseudomonas aeruginosa*, X-ray crystallography, anti-CRISPR, type I-E CRISPR-Cas

## Abstract

CRISPR (clustered regularly interspaced short palindromic repeat)-Cas adaptive immune systems are prevalent defense mechanisms in bacteria and archaea. They provide sequence-specific detection and neutralization of foreign nucleic acids such as bacteriophages and plasmids. One mechanism by which phages and other mobile genetic elements are able to overcome the CRISPR-Cas system is through the expression of anti-CRISPR proteins. Over 20 different families of anti-CRISPR proteins have been described, each of which inhibits a particular type of CRISPR-Cas system. In this work, we determined the structure of type I-E anti-CRISPR protein AcrE1 by X-ray crystallography. We show that AcrE1 binds to the CRISPR-associated helicase/nuclease Cas3 and that the C-terminal region of the anti-CRISPR protein is important for its inhibitory activity. We further show that AcrE1 can convert the endogenous type I-E CRISPR system into a programmable transcriptional repressor.

## IMPORTANCE

The CRISPR-Cas immune system provides bacteria with resistance to invasion by potentially harmful viruses, plasmids, and other foreign mobile genetic elements. This study presents the first structural and mechanistic insight into a phage-encoded protein that inactivates the type I-E CRISPR-Cas system in *Pseudomonas aeruginosa*. The interaction of this anti-CRISPR protein with the CRISPR-associated helicase/nuclease proteins Cas3 shuts down the CRISPR-Cas system and protects phages carrying this gene from destruction. This interaction also allows the repurposing of the endogenous type I-E CRISPR system into a programmable transcriptional repressor, providing a new biotechnological tool for genetic studies of bacteria encoding this type I-E CRISPR-Cas system.

## INTRODUCTION

Bacteria and bacteriophages (phages) coexist in a constant battle for survival. As a result, bacteria have evolved a variety of antiphage defense systems such as restriction modification, superinfection exclusion proteins, and abortive infection mechanisms (1, 2). While these systems provide a type of innate immunity, bacteria also possess an adaptive immune system, known as CRISPR (clustered regularly interspaced short palindromic repeats)-Cas. CRISPR and CRISPR-associated (*cas*) genes comprise an adaptive, RNA-guided immune system that arms bacteria with sequence-specific protection against invasion by foreign nucleic acids such as phages and plasmids. CRISPR-Cas systems are broadly distributed across bacteria and archaea. They are grouped into two broad classes encompassing six types based on their phylogeny and mechanisms of activity (3). Class 1 systems (types I, III, and IV) utilize multisubunit Cas protein complexes (e.g., Cascade) for the recognition and cleavage of targeted nucleic acids, while class 2 systems (types II, V, and VI) use a single protein (e.g., Cas9) that provides all of the required activities (3).

The opportunistic human pathogen *Pseudomonas aeruginosa* possesses two active class 1 CRISPR-Cas systems known as type I-E and type I-F (4, 5). These CRISPR-Cas systems function in three main stages: adaptation, expression, and interference. In the adaptation stage, bacteria integrate segments of foreign DNA into their genomic CRISPR locus, comprising tandem units of short, semipalindromic repeats interspaced with invader-derived “spacer” elements (6). The next stage, expression, involves the transcription of the CRISPR locus into a long precursor CRISPR RNA and subsequent processing to single repeat-spacer units known as mature CRISPR RNAs (crRNAs). A ribonucleoprotein complex called Cascade is then formed by a set of CRISPR-associated (Cas) proteins and crRNA (7). The final stage, interference, occurs when Cascade surveys the cell and identifies target DNA molecules by complementary base pairing with the crRNA spacer (8). This allows recruitment of the Cas3 helicase/nuclease protein that destroys the foreign DNA molecule (9).

While the widespread nature of CRISPR-Cas systems is expected to provide a significant barrier to horizontal gene transfer, studies have shown that they have minimal inhibitory effect over evolutionary timescales (10). One explanation for this could be the presence of small protein inhibitors of CRISPR-Cas systems (11). These anti-CRISPR proteins have been described in association with type I-F, type I-E, type II-C, and type II-A CRISPR-Cas systems in diverse Gram-negative and Gram-positive bacteria (4, 5, 12–15). Recently, structures of three type I-F anti-CRISPR proteins were reported, revealing that each anti-CRISPR protein family possesses a unique fold and interacts with different parts of the CRISPR-Cas machinery (16–20). AcrF1 was shown to bind to the Cas7f hexamer and allosterically inhibit DNA binding, AcrF2 bound Cas8f and acted as a double-stranded DNA mimic that competes with the target DNA for the critical DNA-binding site, and AcrF3 bound Cas3, the effector nuclease and helicase protein common to all type I systems (3). Studies of type II anti-CRISPR proteins revealed two additional unique protein folds and interactions with Cas9 proteins. AcrIIC1 was shown to bind to the HNH endonuclease domain and prevent target DNA cleavage (21), while AcrIIA4 binds the protospacer-adjacent-motif (PAM)-interacting region and blocks interaction with the target DNA molecule (22–24). These studies highlight the structural and mechanistic diversity by which anti-CRISPR proteins function. To date, no structure has been determined for any type I-E anti-CRISPR protein.

Here, we report the 2.5-Å-resolution crystal structure of anti-CRISPR protein AcrE1, which inhibits the type I-E CRISPR-Cas system of *P. aeruginosa*. We have identified a functional region of this protein and show that AcrE1 binds to and inactivates the Cas3 nuclease. Exploiting the ability of AcrE1 to inactivate Cas3, we developed a system where expression of AcrE1 and a promoter-targeting crRNA can convert endogenous type I-E CRISPR-Cas activity into a programmable transcriptional repression system in *P. aeruginosa*.

## RESULTS

### The structure of AcrE1.

We previously reported that gene *34* from *Pseudomonas* phage JBD5, which encodes the protein AcrE1, shows anti-CRISPR activity against the type I-E system of *P. aeruginosa* strain SMC4386 (4). Expression of *acrE1* from a plasmid or prophage allowed infection by a CRISPR-targeted phage and efficient transformation of cells with a CRISPR-targeted plasmid. To gain insight into the mechanism of activity of AcrE1, we solved its three-dimensional structure by X-ray crystallography. Using single-wavelength anomalous diffraction (Br-SAD), we were able to determine the structure of AcrE1 with a C-terminal 6×His tag (PDB identifier [ID] 6ARZ) to a resolution of 2.5 Å (Table 1).

**TABLE 1:** Data collection and refinement statistics for *Pseudomonas* phage protein AcrE1 crystal structures solved in this study

Parameter^a^	CHis AcrE1	NHis AcrE1
PDB code	6ARZ	6AS4
Phasing method	Br-SAD	MR
Data collection		
Space group	C 1 2 1	C 1 2 1
Cell dimensions		
*a*, *b*, *c* (Å)	98.14, 63.96, 59.70	96.74, 63.53, 59.46
a, b, c (°)	90, 100.94, 90	90, 100.34, 90
Solvent content (%)	52.30	45.30
Wavelength (Å)	0.9199	0.9793
Resolution (Å)	48.18–2.5 (2.59–2.50)	47.59–2.0 (2.07–2.0)
*I*/σ*I*	24.1 (7.2)	16.25 (1.57)
Completeness (%)	100 (100)	98.57 (97.59)
Redundancy	7.7 (7.6)	7.7 (7.7)
*R*_merge_	0.06 (0.296)	0.1033 (1.596)
CC_1/2_	0.99 (0.98)	0.999 (0.529)
Refinement		
Resolution (Å)	48.18–2.5	47.59–2.0
No. of unique reflections	12,710 (1,257)	23,742 (2,349)
*R*_work_/*R*_free_	0.18/0.22	0.19/0.23
No. of atoms	2,358	2,415
Protein	282	279
Ligands	30	0
Water	105	150
B-factors		
Protein	58.90	47.30
Ligands	71.10	0
Water	52.30	45.30
RMSD		
Bond length (Å)	0.012	0.008
Bond angle (°)	1.290	0.93
Ramachandran		
Favored (%)	97.10	100
Outlier (%)	0	0

a Highest-resolution shell values are shown in parentheses. R factors were calculated using the formula Σ|*F*_obs_ − *F*_calc_|/Σ*F*_obs_. CC_1/2_ was calculated based on Pearson’s correlation coefficient, and the cutoff value was 0.5.

**FIG 1:**
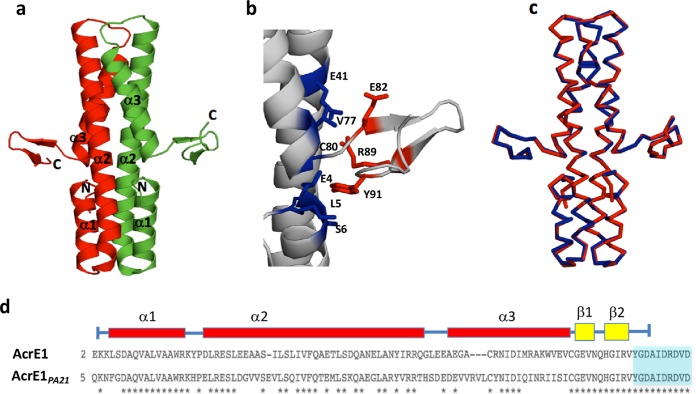
Structure of AcrE1. (a) Crystal structure of the C-terminal 6×His-tagged AcrE1 solved by Br-SAD reveals a predominantly helical dimeric protein. The N and C termini are noted. (b) Interactions between residues in the AcrE1 β-strands (colored red) and residues in the helices (colored blue) stabilize the C-terminal β-sheet. At least 10% of the surface area of each residue shown is buried by the interaction between the helices and the C-terminal region. A salt bridge is formed between E41 and R89. (c) Structural overlay of the X-ray crystal structures of the AcrE1 proteins with an N-terminal (blue) and C-terminal (red) 6×His tag reveals virtually identical structures. (d) Sequence alignment of the two homologues of the AcrE1 protein family with validated activities against the type I-E CRISPR-Cas system. Asterisks indicate identical residues shared between the two proteins. Blue shading indicates the amino acids absent from the AcrE1^Δ91–100^ truncation mutant.

AcrE1 displays an elongated dimeric structure (Fig. 1a). Each monomer is predominantly comprised of three helices. Helix 1 (residues 5 to 17) is followed by a tight turn leading into the very long helix 2 (residues 21 to 57). A short turn at the end of helix 2 leads into helix 3 (residues 62 to 80), which terminates close enough to the beginning of helix 1 for a potential H-bond to form between the carbonyl O of Val79 and the amide N of Leu5. Residues 82 to 90 form an antiparallel β-sheet that protrudes at a 90° angle from the end of helix 3. This sheet structure appears to be stabilized by multiple interactions between residues at the base of the sheet and residues in helices 1, 2, and 3 (Fig. 1b). The final 10 residues of AcrE1 are likely disordered, as they were not well defined in the electron density map and could not be modeled into the structure. The large dimeric interface is composed primarily of 18 hydrophobic residues from each monomer that are greater than 97% buried, forming a single hydrophobic core that spans the entire length of the helices (see Fig. S1a in the supplemental material). The dimeric crystal structure of AcrE1 is consistent with the native molecular weight of this protein in solution as estimated by size exclusion chromatography (SEC) (Fig. S1b).

10.1128/mBio.01751-17.1 FIG S1  (a) AcrE1 has a well-packed hydrophobic core that spans the length of the helices. Each monomer buries 18 hydrophobic residues in the dimeric interface. (b) Estimation of the native molecular weight of AcrE1 CHis in solution. The elution volume of the single peak observed after size exclusion chromatography of purified AcrE1 CHis was used with the standard curve to calculate an estimated molecular mass of 24.41 kDa, corresponding closely to the theoretical molecular mass of a dimer of the 100-amino-acid AcrE1 protein. Download FIG S1, TIF file, 2.6 MB. Copyright © 2017 Pawluk et al. 2017 Pawluk et al. This content is distributed under the terms of the Creative Commons Attribution 4.0 International license.

The structure of AcrE1 is not similar to other previously determined anti-CRISPR proteins, and searches using the Dali server (25) did not reveal structural similarity to any other protein. The C-terminal β-sheet region is unusual because both sides of the sheet are exposed to solvent, contrasting with typical β-sheets, which pack a hydrophobic surface on one side against helices or other sheets. To ensure that this structure was not a result of the presence of the C-terminal 6×His tag, we also crystalized AcrE1 with an N-terminal 6×His tag (PDB ID 6AS4) and determined its structure using molecular replacement (MR) to a resolution of 2.0 Å (Table 1). This structure was virtually identical to the C-terminally tagged structure; the two structures could be aligned over 736 backbone atoms with a root mean square deviation (RMSD) of 0.31 Å (Fig. 1c). Thus, the unusual nature of the C-terminal sheet was not a result of the presence of the 6×His affinity purification tag fused to the C terminus. We also examined the packing of the C-terminal β-sheet within the crystal and observed no contacts that stabilize it in this position (Fig. S3).

### Identification of functionally critical surfaces of AcrE1.

To identify amino acid residues in AcrE1 that are required for its function, we combined structural and sequence analyses with site-directed mutagenesis. A homologue of AcrE1 encoded by a conjugative element in *P. aeruginosa* that shares 62% sequence identity (AcrE1_*PA21*_) was previously found to also block type I-E CRISPR-Cas activity in *P. aeruginosa* strain SMC4386 (4). We identified 20 residues that were exposed on the surface of AcrE1 (accessible surface area, ≥35%) that were largely conserved between the two proteins and introduced mutations at each of these positions (Fig. S2a and b). Each mutant protein was expressed from a plasmid in strain SMC4386, and its ability to support replication of a CRISPR-targeted phage was assessed. None of the AcrE1 mutants tested showed any reduction in anti-CRISPR activity as measured by this assay. These results contrast with a study on anti-CRISPR protein AcrF1, in which three different single-site substitutions on one surface of the protein caused a marked inhibition of anti-CRISPR activity (16).

10.1128/mBio.01751-17.2 FIG S2  (a) Amino acid substitutions were engineered at 20 positions exposed on the surface of AcrE1, and the biological effects of these mutations were assessed using an *in vivo* phage plaquing assay. (b) The 20 amino acid substitutions mapped onto the structure of AcrE1. Download FIG S2, TIF file, 2.6 MB. Copyright © 2017 Pawluk et al. 2017 Pawluk et al. This content is distributed under the terms of the Creative Commons Attribution 4.0 International license.

10.1128/mBio.01751-17.3 FIG S3  (a) Crystal packing of AcrE1. (b) Crystal contacts showing the region around the C terminus of AcrE1. There are no crystal contacts that stabilize the C terminus observed. Download FIG S3, TIF file, 2.6 MB. Copyright © 2017 Pawluk et al. 2017 Pawluk et al. This content is distributed under the terms of the Creative Commons Attribution 4.0 International license.

**FIG 2:**
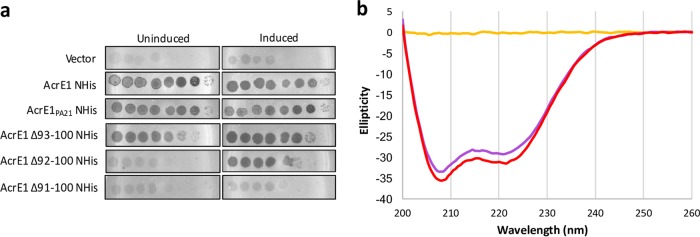
Deletion of 10 C-terminal residues from AcrE1 results in a complete loss of activity, while a significant proportion of soluble, folded protein is retained. (a) Tenfold serial dilutions of CRISPR-targeted phage JBD8 were spotted on bacterial lawns of *P. aeruginosa* strain SMC4386, and the ability to replicate in the presence of an active type I-E CRISPR system was assessed. The bacteria were expressing wild-type AcrE1, the homologue of AcrE1 encoded by a conjugative element in *Pseudomonas* strain PA21, or one of the deletion mutants from a plasmid as noted on the left. Bacteria with low-level expression of AcrE1 (uninduced, left panels) or high-level expression (induced, right panels) were challenged with phage. Zones of clearing indicate phage replication. The replication efficiency of a CRISPR-insensitive phage, JBD93a, remained constant on all strains (data not shown). Images are representative of three independent experiments. (b) Circular dichroism (CD) spectra of AcrE1 NHis (purple) and AcrE1^Δ91–100^ NHis (red) reveal very similar spectra with canonical α-helical signals. The spectrum of the buffer (yellow) is provided for reference. The spectra shown here are representative of three independent experiments.

Further comparison of the two AcrE1 homologues revealed a striking pattern of conservation: their helical regions (residues 1 to 79) share 54% sequence identity, while their C-terminal regions (residues 80 to 100) share 100% identity (Fig. 1d). This completely conserved C-terminal region encompasses the short β-sheet and the 10 residues at the extreme C terminus that do not form a defined secondary structure. To delineate the functional importance of this C-terminal region, we created a series of C-terminal truncation mutants and assessed the anti-CRISPR activity of each. We found that the mutant with eight C-terminal residues (AcrE1^Δ93–100^) deleted had wild-type levels of anti-CRISPR activity (Fig. 2a). The mutant with nine residues truncated (AcrE1^Δ92–100^) displayed no anti-CRISPR activity. However, this activity could be almost completely recovered through addition of arabinose, which strongly induces the plasmid-borne transcriptional promoter (Fig. 2a). This suggests either that AcrE1^Δ92–100^ is less stable and accumulates to lower steady-state levels in the cell or that it has reduced binding affinity for its target, such that a large excess of the protein is able to restore functionality. Deletion of one additional residue (AcrE1^Δ91–100^) led to complete loss of activity, even in the presence of arabinose (Fig. 2a).

**FIG 3:**
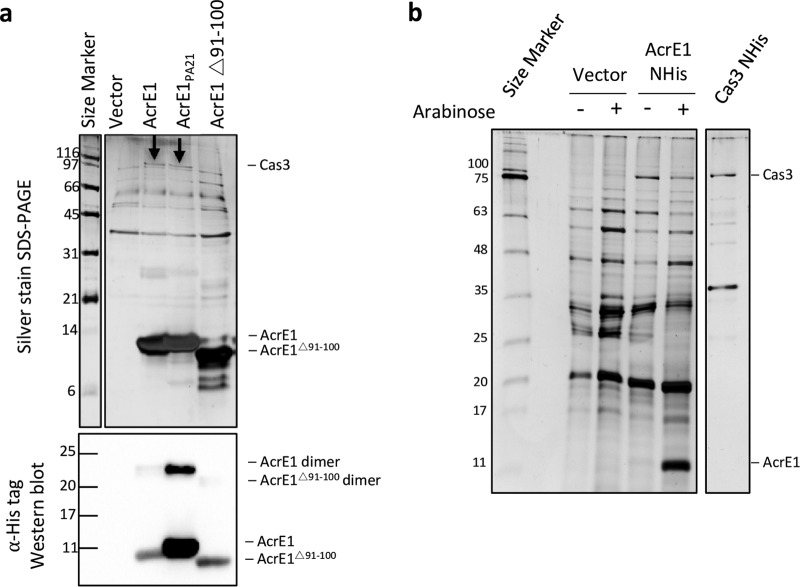
AcrE1 interacts with the type I-E CRISPR-Cas protein, Cas3. (a) N-terminal 6×His-tagged AcrE1, AcrE1_*PA21*_, or AcrE1^Δ91–100^ was expressed from a plasmid in *P. aeruginosa* SMC4386 cells. Following *in vivo* formaldehyde cross-linking, anti-CRISPR proteins were affinity purified using Ni-NTA agarose beads, and the resultant elutions were analyzed by SDS-PAGE followed by silver staining (upper panel) and anti-His Western blotting (lower panel). AcrE1_*PA21*_ accumulates to higher steady-state levels in the cell than AcrE1 and AcrE1^Δ91–100^. (b) Low levels of AcrE1 bind intracellular Cas3 to saturation. Purified 6×His-tagged Cas3 expressed from a plasmid in SMC4385 cells was run as a size standard for the Cas3 protein (far right lane). Even very low levels of AcrE1 were sufficient to pull down as much Cas3 as the overexpressed versions, suggesting that all the Cas3 in the cells is saturated by anti-CRISPR protein binding. Four irrelevant lanes were removed from the gel image. Numbers at left of each panel are molecular masses in kilodaltons.

To determine if the loss of function of AcrE1^Δ91–100^ was a result of lack of accumulation in the cell due to expression defects or proteolysis, we assessed intracellular protein levels. As can be seen in Fig. 3a, wild-type AcrE1 and AcrE1^Δ91–100^ could be purified from cells at comparable levels when expressed from a plasmid in *P. aeruginosa* SMC4386. We purified these two proteins from *Escherichia coli* for further analyses. Similar amounts of wild-type AcrE1 and AcrE1^Δ91–100^ were recovered, and circular dichroism (CD) spectroscopy revealed very similar spectra with a canonical α-helical signature (Fig. 2b). While wild-type AcrE1 was soluble to a high concentration, AcrE1^Δ91–100^ was less soluble and formed aggregates *in vitro* upon storage, and so no further structural work was done with this mutant. The circular dichroism data indicated that that AcrE1^Δ91–100^ was folded and that there were no gross changes in the secondary structure content of this truncated protein. Thus, it is likely that residues 91 and 92 of AcrE1 are involved in protein-protein interaction(s) necessary for its anti-CRISPR activity.

### AcrE1 interacts with Cas3.

To identify the protein interaction partners of AcrE1, we performed copurification experiments. *P. aeruginosa* strain SMC4386 was transformed with plasmids expressing AcrE1, AcrE1_*PA21*_, or AcrE1^Δ91–100^. After induction of protein expression from the plasmids, cells were exposed to formaldehyde to cross-link protein complexes *in vivo* (26). Chemical cross-linker was used in these experiments because the endogenous expression level of Cas proteins in this strain was low, and we aimed to maximize capture of AcrE1-Cas protein complexes. The 6×His-tagged AcrE1 proteins were purified by nickel affinity chromatography, and copurifying proteins were analyzed by SDS-PAGE followed by silver staining. In this way, we identified a distinct band at approximately 97 kDa that copurified with AcrE1 and AcrE1_*PA21*_ but not with the inactive C-terminal truncation mutant, AcrE1^Δ91–100^ (Fig. 3a). This band was excised and analyzed using mass spectrometry, leading to the identification of 55 unique peptides from Cas3 covering 67% of the protein (Tables S1 and S2). The type I-E Cas3 protein of *P. aeruginosa* has a calculated molecular mass of 99 kDa, in agreement with the apparent molecular mass observed on the protein gel. Thus, we concluded that AcrE1 binds to Cas3, potentially preventing its recruitment to the DNA-bound Cascade complex.

We previously showed that arabinose induction of the plasmid promoter that drives AcrE1 expression was not necessary for full anti-CRISPR activity (4). This result demonstrated that a small amount of anti-CRISPR protein is sufficient to completely block the CRISPR-Cas system. In support of this, we saw no increase in the level of Cas3 copurifying with AcrE1 in the presence of arabinose compared to the uninduced control (Fig. 3b), suggesting that all the Cas3 present in cells is bound to AcrE1 in both cases.

10.1128/mBio.01751-17.4 TABLE S1  LC-MS/MS results from the prominent ~97-kDa band excised from an SDS-PAGE gel after affinity purification of AcrE1 NHis from *P. aeruginosa* SMC4386 cells. The peptides identified were mapped to the *P. aeruginosa* PA2192 proteome. The table lists the top 10 hits sorted by percent coverage. Download TABLE S1, DOCX file, 0.1 MB. Copyright © 2017 Pawluk et al. 2017 Pawluk et al. This content is distributed under the terms of the Creative Commons Attribution 4.0 International license.

10.1128/mBio.01751-17.5 TABLE S2  Normalized total spectral counts for elution fractions from native nickel affinity purification of formaldehyde cross-linked samples. The 6×His-tagged anti-CRISPR protein expressed in *P. aeruginosa* SMC4386 cells for each sample is indicated at the top. Cross-links were reversed using heat prior to LC-MS/MS analysis, and peptides were mapped to the proteome of *P. aeruginosa* strain PA2192. This table contains a selected list of hits with high numbers of peptides and high confidence of protein identification. Abundant proteins and common contaminants are detected similarly across the three samples, but only full-length AcrE1 copurifies with Cas3. Download TABLE S2, DOCX file, 0.1 MB. Copyright © 2017 Pawluk et al. 2017 Pawluk et al. This content is distributed under the terms of the Creative Commons Attribution 4.0 International license.

### AcrE1 blocks the activity of Cas3.

**FIG 4:**
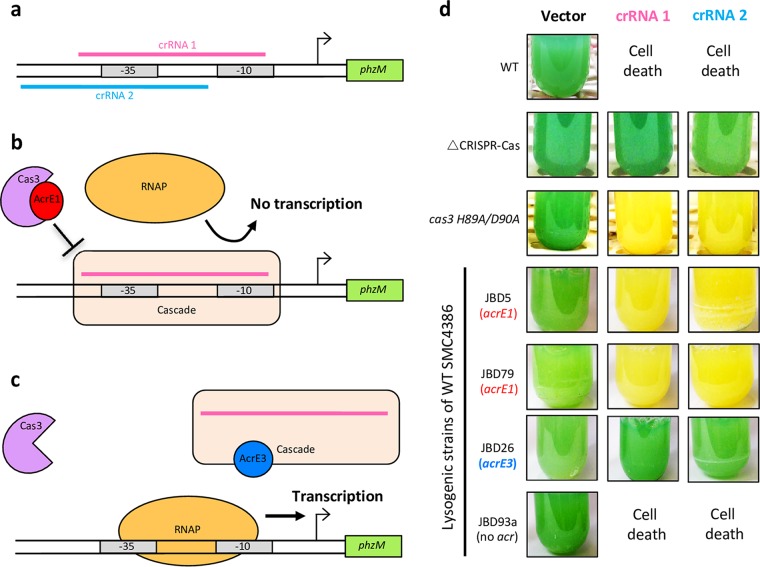
Cas3 inactivation converts the *P. aeruginosa* type I-E CRISPR-Cas system into a programmable transcriptional repression platform. (a) crRNA1 and crRNA2 target the promoter region upstream of the *phzM* gene. (b) Conceptual model for transcriptional repression by Cascade targeted to the *phzM* promoter, in the case where an anti-CRISPR protein inactivates or prevents the recruitment of Cas3. The targeting of Cascade to the promoter region upstream of *phzM* would prevent access by RNA polymerase (RNAP), thereby locking transcription of the gene. The same outcome would result from a *cas3* deletion or inactivating mutation. (c) A conceptual model for the normal transcription of *phzM* resulting from the presence of an anti-CRISPR protein that blocks Cascade from binding to target DNA. The same outcome would be expected for a CRISPR-Cas deletion strain, in which the Cascade complex is absent. (d) Photographs of *P. aeruginosa* SMC4386 expressing crRNA1, crRNA2, or empty-vector control. In the presence of the wild-type CRISPR-Cas system (WT), cell death occurs due to self-targeting of the chromosome by crRNA1 and crRNA2. In the absence of an active CRISPR-Cas system (ΔCRISPR-Cas), normal transcription of *phzM* occurs, resulting in a green pigmentation. A Cas3 mutant (H89A/D90A) leads to loss of the green pigment, indicating that the Cascade complex is repressing expression of *phzM*. The expression of AcrE1 from prophages JBD5 and JBD79 also leads to repression of *phzM* expression, as indicated by the loss of green pigment. In contrast, a prophage with no anti-CRISPR protein (JBD93a) or a different type I-E anti-CRISPR protein (JBD26; AcrE3) led to cell death and normal pyocyanin production, respectively.

In the absence of Cas3, Cascade can still bind DNA strongly and specifically as guided by its crRNA. Therefore, if Cascade is targeted to a promoter in the absence of Cas3, transcription is repressed as the bound Cascade complex prevents recruitment of RNA polymerase (27–29). To determine whether AcrE1 inhibits the activity of Cas3 *in vivo*, we designed crRNA1 and crRNA2, which targeted protospacers in the −10 and −35 regions of the promoter of *phzM*, a biosynthetic gene essential for the production of the blue-green pigment pyocyanin (Fig. 4a). In this assay, an anti-CRISPR protein that blocks the recruitment of Cas3 to the Cascade complex will result in loss of pyocyanin production due to the targeted Cascade complex blocking access of RNA polymerase (Fig. 4b). By contrast, expression of an anti-CRISPR protein that inhibits Cascade from binding to its DNA target results in normal pyocyanin production (Fig. 4c). When strain SMC4386 was transformed with plasmids expressing crRNA1 or crRNA2, cell death resulted from CRISPR-Cas targeting of the bacterial genome. However, introduction of these plasmids into the same strain containing a JBD5 or JBD79 prophage, which both express AcrE1, did not cause cell death due to the AcrE1-mediated anti-CRISPR activity. In addition, overnight cultures of these strains were completely devoid of green pigmentation, implying that the targeted *phzM* promoter was being repressed (Fig. 4b and d). This same behavior was observed when the experiment was performed with a strain expressing an inactive Cas3 protein, demonstrating that expression of AcrE1 phenocopies a strain lacking Cas3 activity. Expression of the crRNAs in an SMC4386 strain with its CRISPR-Cas locus completely deleted resulted in green pigmentation, as no Cascade complex was present to mediate *phzM* repression. All strains transformed by empty vector also displayed bright green pigmentation. Introduction of the crRNA-expressing plasmids into wild-type SMC4386 bearing a JBD26 prophage, which expresses a different type I-E anti-CRISPR protein (AcrE3) (4), resulted in green pigmentation, suggesting that this anti-CRISPR protein blocks Cascade DNA binding (Fig. 4c and d). In summary, these experiments demonstrate that AcrE1 functions by blocking the activity of Cas3 without inhibiting the DNA-binding activity of Cascade. AcrE3 elicits a contrasting behavior and likely abrogates the DNA-binding activity of Cascade. Thus, AcrE1 can convert the activity of the CRISPR-Cas interference complex into a gene regulatory function.

## DISCUSSION

The evolutionary arms race between bacteria and phages provides a strong selective pressure for the emergence and maintenance of anti-CRISPR proteins. Consistent with this selective pressure, 14 diverse families of anti-CRISPR inhibitors of the *P. aeruginosa* type I-E or I-F CRISPR-Cas system have been described (4, 5, 13). In this work, we determined the first structure of a type I-E anti-CRISPR protein. To date, structures have been determined for three *P. aeruginosa* type I-F anti-CRISPR proteins (16–20), a type II-A protein from *Listeria monocytogenes* (22–24), and a type II-C protein from *Neisseria meningitidis* (21). The structure of AcrE1 does not resemble any of these previously determined structures and thus provides the sixth unique structural family of proteins that can inactivate CRISPR-Cas systems. This wide structural diversity provides further support for the independent evolution of anti-CRISPR protein families as previously proposed (29).

Type I CRISPR-Cas systems are defined by the presence of the gene *cas3*, which encodes an ~100-kDa protein with single-stranded DNA nuclease and ATP-dependent helicase activities (3). Cas3 is not part of the Cascade complex but is recruited to degrade the target DNA after Cascade binds to it (9). We found that AcrE1 binds to Cas3 using copurification assays (Fig. 3), and the *phzM* transcriptional repression assays showed that this binding blocks Cas3 activity (Fig. 4). We previously showed that anti-CRISPR AcrF3 blocks activity of the type I-F CRISPR-Cas system of *P. aeruginosa* through an interaction with Cas3 (29), and we expect that AcrE1 works in a similar manner. Although the sequences of Cas3 proteins from type I-E and I-F systems are highly diverse, their structures are very similar (18). Thus, it is interesting that AcrE1 and AcrF3 display very different folds. They clearly bind Cas3 through distinct mechanisms, either targeting different surfaces on the Cas3 structure or utilizing different binding modes to target the same surface.

We performed an extensive mutagenesis study covering all of the conserved residues with side chains exposed on the surface of AcrE1 (see Fig. S2 in the supplemental material). All single-residue replacements displayed wild-type anti-CRISPR activity, with only the C-terminal truncation of 10 amino acids causing complete loss of activity. These results imply that the C terminus of AcrE1 forms the crucial interactions with Cas3 that lead to its inhibition. These results mirror those that we obtained in a previous study on AcrF1 in which substitutions at only 3 out of 35 surface-exposed residues tested caused a significant reduction in anti-CRISPR activity. In both of these studies, the anti-CRISPR proteins appear to utilize a very small functional interface as defined by mutagenesis. In the case of AcrF1, however, the functional interface defined by mutagenesis is considerably smaller than the interaction interface between AcrF1 and the CRISPR-Cas complex observed in the co-complex structure determined by cryo-electron microscopy (17). Thus, we cannot conclude from our mutagenesis that AcrE1 forms a small interaction interface with Cas3. In this regard, it is notable that the type I-F anti-CRISPR protein, AcrF3, forms a very large interaction interface with Cas3 (18, 19), comprising an area of ~2,500 Å^2^. The requisite mutagenesis studies have not yet been performed to delineate the functional interface of this complex, which may also turn out to be much smaller than indicated by the structure.

The ability of AcrE1 to inhibit Cas3 activity allowed us to convert the endogenous type I-E CRISPR-Cas system into a specific repressor of the *phzM* promoter. This experiment proved that AcrE1 inhibits Cas3 and also demonstrated the potential for a CRISPR-based tool for transcriptional repression of target genes in bacteria. Any strain possessing an active CRISPR-Cas system that can be inhibited by either AcrE1 for type I-E or AcrF3 for type I-F (29) can be transformed with a single plasmid expressing anti-CRISPR protein and a promoter-targeting crRNA to elicit specific gene repression. Anti-CRISPR proteins from *P. aeruginosa* have proven effective at inhibiting the type I-F CRISPR-Cas system of *Pectobacterium atrosepticum*, whose Cas proteins share less than 50% pairwise sequence identity to *P. aeruginosa* (13), indicating that the transcriptional repression system may work in many diverse bacterial lineages. Due to the small size of the crRNA molecules for these systems, targets can easily be synthesized as oligonucleotides and cloned into an anti-CRISPR protein-expressing plasmid, enabling quick, cost-effective, multiplexable, and robust transcriptional repression of endogenous bacterial gene expression.

Our AcrE1-mediated transcriptional repression results raise the possibility that anti-CRISPR proteins could be co-opted in some species to modulate CRISPR-Cas activity for the purpose of gene regulation. It is possible that both bacteria and phages encoding Cas3-targeting anti-CRISPR proteins could benefit from Cascade-mediated transcriptional control. For example, an AcrE1-encoding phage could encode a crRNA targeting one or more host promoters in order to silence genes involved in phage resistance or to otherwise modulate host transcription in ways that would benefit the phage. It is also possible that bacteria with CRISPR-Cas systems could subvert the activity of this anti-CRISPR protein and target a phage promoter to silence essential phage genes and prevent it from entering into the viral replicative cycle.

Twenty-two distinct anti-CRISPR protein families have been discovered to date that inhibit four different types of CRISPR-Cas systems (4, 5, 12–15). Although relatively few have been studied in detail, it is clear that phages utilize several different strategies to inhibit the CRISPR-Cas machinery. Further structural and biochemical characterization of anti-CRISPRs will illuminate the full spectrum of inhibition mechanisms and will provide deeper insight into the functioning of diverse CRISPR-Cas systems.

## MATERIALS AND METHODS

### Bacterial growth and phage propagation.

*Pseudomonas aeruginosa* was grown in LB liquid medium at 37°C with shaking or on LB agar plates at 37°C overnight, unless otherwise indicated. When necessary, media were supplemented with 50 μg/ml gentamicin to maintain the pHERD30T plasmid (30). Plasmids were introduced into *P. aeruginosa* SMC4386 by electroporation. Induction of the plasmid promoter for overexpression of anti-CRISPR genes was achieved by supplementing medium with 4 mM arabinose.

Phages were propagated by mixing with *P. aeruginosa* SMC4386 Δ*CRISPR-cas*, a susceptible host. The phage-host mixture was added to LB containing 0.7% agar and 10 mM MgSO_4_ and poured onto thick LB agar (1.5%) plates containing 10 mM MgSO_4_. After overnight incubation at 30°C, plates with near-confluent lysis were soaked with SM buffer for 3 h for phage extraction, followed by centrifugation at 14,000 rpm to remove cell and agar debris. Phages were stored in SM buffer over chloroform at 4°C.

### Phage plaque assays.

To measure type I-E CRISPR-Cas activity in *P. aeruginosa* SMC4386, phage plaque assays were conducted as described previously (4). *P. aeruginosa* SMC4386 cells containing pHERD30T plasmid expressing AcrE1 were mixed with 0.7% LB agar and overlaid onto LB agar (1.5%) plates containing 10 mM MgSO_4_, 50 μg/ml gentamicin, and (where indicated) 4 mM arabinose to induce anti-CRISPR gene expression. Tenfold serial dilutions of a CRISPR-targeted phage (JBD8) and a CRISPR-insensitive phage (JBD93a) were spotted on the surface, and the plates were incubated overnight at 30°C.

### Site-directed mutagenesis.

Pairs of complementary oligonucleotides containing the codon to be mutated plus five codons on either side were synthesized by Eurofins Genomics. *Pfu* DNA polymerase was used to PCR amplify DNA from template plasmid pHERD30T containing the *acrE1* gene. After template digestion with DpnI (New England BioLabs [NEB]), the DNA was concentrated and purified by ethanol precipitation and used to transform *E. coli* DH5α. Mutations were confirmed by DNA sequencing, and mutant plasmids were introduced into *P. aeruginosa* SMC4386 for phage plaque assays.

### AcrE1 expression and protein purification.

The AcrE1 open reading frame was cloned by ligation into pET21d(+) with either an N-terminal (NHis) or C-terminal (CHis) noncleavable 6×His tag. *E. coli* BL21(DE3) cells carrying either plasmid maintained in 100 µg/ml ampicillin were grown to an optical density at 600 nm (OD_600_) of 0.5 to 0.6, and IPTG (isopropyl-β-d-thiogalactopyranoside) was added to a final concentration of 0.8 mM. After 4 h of shaking incubation at 37°C, cells were pelleted and resuspended in binding buffer (20 mM Tris-HCl, pH 7.5, 200 mM NaCl, 5 mM β-mercaptoethanol) with 5 mM imidazole added. Cells were lysed by sonication, and lysates were cleared by centrifugation for 20 min at 17,000 rpm. Nickel-nitrilotriacetic acid (Ni-NTA) beads (Qiagen) were incubated with the clarified lysate for 30 min at 4°C. Column purification at room temperature was performed with washes in wash buffer (binding buffer plus 30 mM imidazole), and the protein was eluted in elution buffer (binding buffer plus 300 mM imidazole). Binding buffer was used for overnight dialysis at room temperature. The protein was further purified by size exclusion chromatography (SEC) using either a Superdex 200 16/60 column (for large-scale purification) or a Superdex 75 10/30 column (for analytical SEC prior to CD spectroscopy) in binding buffer. The same purification methods and buffers were used to purify AcrE1^Δ91–100^ NHis. Selenomethionine (Se-Met)-labeled CHis AcrE1 was expressed using the methionine-auxotrophic *E. coli* BL21(DE3) B834 strain cultured in M9 minimal medium containing 0.2% glucose and trace metals supplemented with Se-Met and was purified using the same protocol as described above.

### Crystallization of AcrE1.

Purified AcrE1 CHis or NHis (native) was initially screened with a 1:1 (protein/precipitant) ratio against the MCSG commercial suite and JCSG+ commercial screen using sitting drop vapor diffusion at 10 mg/ml. AcrE1 CHis crystals were observed in 0.1 M sodium cacodylate buffer, pH 6.0, 0.1 M NaBr, and 25% polyethylene glycol (PEG) 3350. The crystals were further optimized with a 1:1-ratio sitting drop at 20°C under a precipitant condition composed of 0.07 M sodium cacodylate, pH 6.1, 0.5 M NaBr, 27% PEG 3350, and 15% glycerol, yielding single crystals in space group C2. Native NHis AcrE1 crystals were observed in 0.2 M ammonium citrate dibasic, 20% (wt/vol) PEG 3350. The crystals were further optimized with a 1:1-ratio sitting drop at 20°C under a precipitant condition composed of 0.2 M ammonium citrate dibasic, pH 7.0, 25% (wt/vol) PEG 3350, and 15% glycerol, yielding single crystals in space group C2.

### Data collection and structure determination.

CHis and NHis AcrE1 crystallographic data were collected on crystals frozen at 105 K on 08B1-1 and 08ID-1 beamlines at Canadian Light Source (CLS), respectively. Diffraction data from a total of 360 images were collected at wavelengths of 0.9199 and 0.9795 using 1° oscillations. Data were processed with the XDS package to a resolution of 2.5 and 2.0 Å for CHis and NHis AcrE1, respectively. A complete model for CHis was solved by Br-SAD with additional anomalous signal from Se atoms using Phenix AutoSol. The final structure was obtained after multiple rounds of refinements and building cycles using Phenix Refine and Coot software packages, yielding a final *R*_work_/*R*_free_ of 0.18/0.22. The structure of NHis AcrE1 was obtained by molecular replacement using CHis as a starting model using Phaser. The final model was generated after several rounds of model building and refinement using Coot and the Phenix Refine program using TLS (translation, liberation, screw), yielding a final *R*_work_/*R*_free_ of 0.18/0.23 for native NHis AcrE1.

### CD spectroscopy.

Circular dichroism (CD) was conducted using an 0.1-cm quartz cuvette at 25°C with a measurement range of 260 to 200 nm. The scanning speed was 50 nm/min with a bandwidth of 1 nm, response time of 2 s, and data pitch of 1.0 nm. Protein concentrations for both wild-type and mutant His-tagged AcrE1 were 16 µM in buffer containing 20 mM Tris-HCl, pH 7.5, 200 mM NaCl, and 5 mM β-mercaptoethanol. Triplicate measurements were recorded and averaged for each CD run. Three biological replicates were collected.

### *In vivo* formaldehyde cross-linking and affinity purification.

One-hundred-milliliter cultures of *P. aeruginosa* strain SMC4386 containing plasmids encoding 6×His-tagged anti-CRISPR AcrE1 or empty vector were grown at 37°C in LB medium. At an OD_600_ of 0.5, 4 mM arabinose was used to induce plasmid expression, except for the uninduced samples in Fig. 3b, to which no arabinose was added. After a 2-h incubation, 200 μl of 37% formaldehyde solution was added, and cells were incubated with shaking for an additional 30 min at 37°C. Glycine was added to a final concentration of 100 mM to quench the cross-linking reaction, and cells were pelleted. One milliliter of Novagen BugBuster reagent was used to resuspend the cell pellet, followed by 1 h of shaking incubation at 25°C and centrifugation at 14,000 rpm for 5 min. We added 50 μl Ni-NTA beads (Qiagen) to each clarified lysate and purified with the same buffer system as described for AcrE1 purification above. One hundred microliters of elution buffer was added to the washed beads and incubated at 100°C for 15 min to elute proteins and reverse heat-labile formaldehyde cross-links (26). Proteins were analyzed by SDS-PAGE followed by silver staining and by SDS-PAGE followed by anti-His-tag Western blotting, as well as by trypsin digest followed by linear trap quadrupole (LTQ) (Thermo Scientific) liquid chromatography-tandem mass spectrometry (LC-MS/MS) (SPARC Biocentre, SickKids, Toronto, Canada).

### Transcriptional repression assay.

Pairs of complementary oligonucleotides were designed to encode a CRISPR repeat-spacer-repeat unit in which the repeat sequence is the *P. aeruginosa* SMC4386 consensus repeat (5′-GTGTTCCCCACATGCGTGGGGATGAACCG-3′) and the spacer sequence matches a 32-nucleotide sequence in the *phzM* promoter element flanked by a consensus protospacer adjacent motif (PAM), 5′-NTT-3′. The sense-strand crRNA sequences (with repeats in lowercase and spacer in uppercase) used in this study are crRNA 1 (5′-gtgttccccacatgcgtggggatgaaccgAATAAAATTACAACTTGGCTACAACCTCCGGCgtgttccccacatgcgtggggatgaaccg-3′) and crRNA 2 (5′-gtgttccccacatgcgtggggatgaaccgCTGATGCTTCCTGCAATGCCGGAGGTTGTAGCgtgttccccacatgcgtggggatgaaccg-3′). Once the oligonucleotides were annealed by heating to 95°C and slow cooling to 10°C, sticky ends were formed that could ligate into pHERD30T predigested with EcoRI and HindIII restriction endonucleases. Ligated plasmids were sequence confirmed and then used to transform SMC4386 cells by electroporation. A dramatic reduction in transformation efficiency of the crRNA-containing plasmids compared to the empty vector indicated that the crRNAs were expressed and processed and successfully targeted the CRISPR-Cas system to the host genome. The indicated mutant or lysogenic strains of SMC4386 were transformed with the crRNA plasmid or empty vector. To ensure that the observed phenotypes were not due to the prophage insertion site (which, for these phages, is random), all results were confirmed using a second, independently isolated lysogen (data not shown). Colonies were used to inoculate King’s A liquid medium (20 g/liter peptone, 10 g/liter potassium sulfate, 1.64 g/liter anhydrous magnesium chloride, and 1% glycerol) supplemented with 50 µg/ml gentamicin to maintain pHERD30T and grown at 37°C. After 10 h of growth, plasmid expression of crRNAs was induced by the addition of 4 mM arabinose, and induction was allowed to proceed for 16 h at 37°C.

### Construction of SMC4386 *cas3* H89A/D90A mutant.

The *cas3* gene was cloned by ligation into pHERD30T (30), and site-directed mutagenesis was performed to introduce the double mutation of H89A and D90A, which comprise the active site residues of the HD nuclease domain of Cas3. The resultant plasmid was used to transform *P. aeruginosa* SMC4386, and cells were grown in LB under 50-μg/ml gentamicin selection for 48 h and then passaged in antibiotic-free LB medium for 72 h to cause plasmid loss. A type I-E CRISPR locus containing spacers targeting highly conserved regions of several essential genes in *P. aeruginosa*, including RNA polymerase and DNA polymerase, was ligated into pHERD20T, a plasmid identical to pHERD30T but with a carbenicillin (instead of gentamicin) resistance cassette. This plasmid was used to select for cells in which recombination of the mutated *cas3* gene had occurred and CRISPR-Cas activity was lost due to the lack of nuclease function. The plasmid should not be able to transform cells with an active CRISPR-Cas system, as they would target and cleave their own genome at several key locations that are, in combination, unescapable by mutation. Sequencing was used to confirm that the correct mutation in *cas3* had caused the loss of CRISPR-Cas activity.
