# Supplementary figures and images for: Disabling a Type I-E CRISPR-Cas Nuclease with a Bacteriophage-Encoded Anti-CRISPR Protein
Source: mBio. 2017 Dec 12;8(6):e01751-17. doi: 10.1128/mBio.01751-17 (PMC5727412; doi:10.1128/mBio.01751-17)

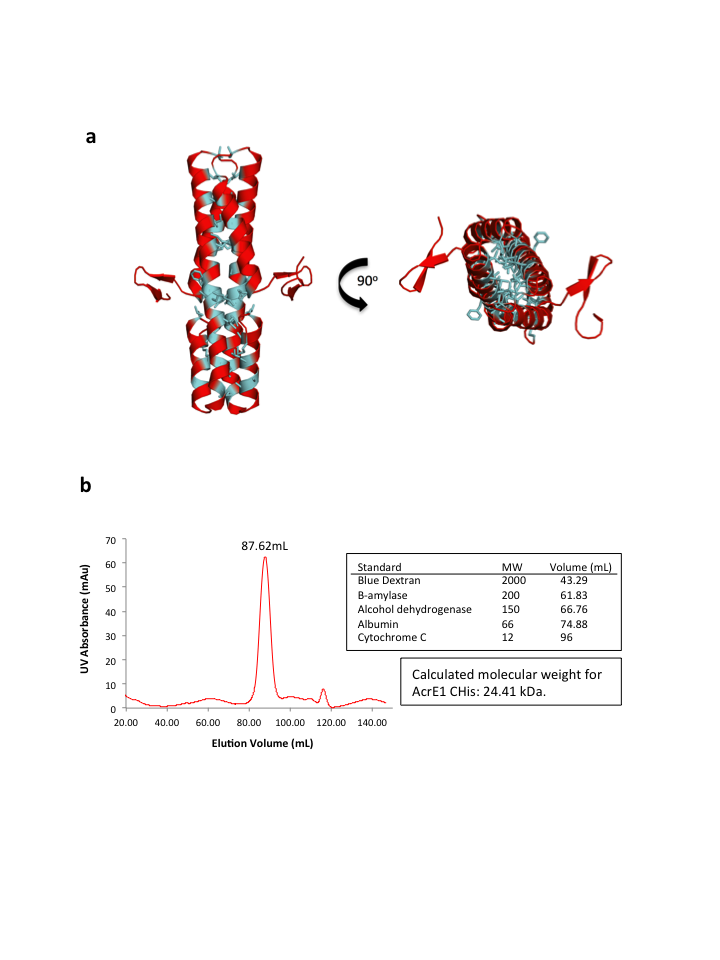

Supplement: FIG S1 [file mbo006173630sf1.tif]

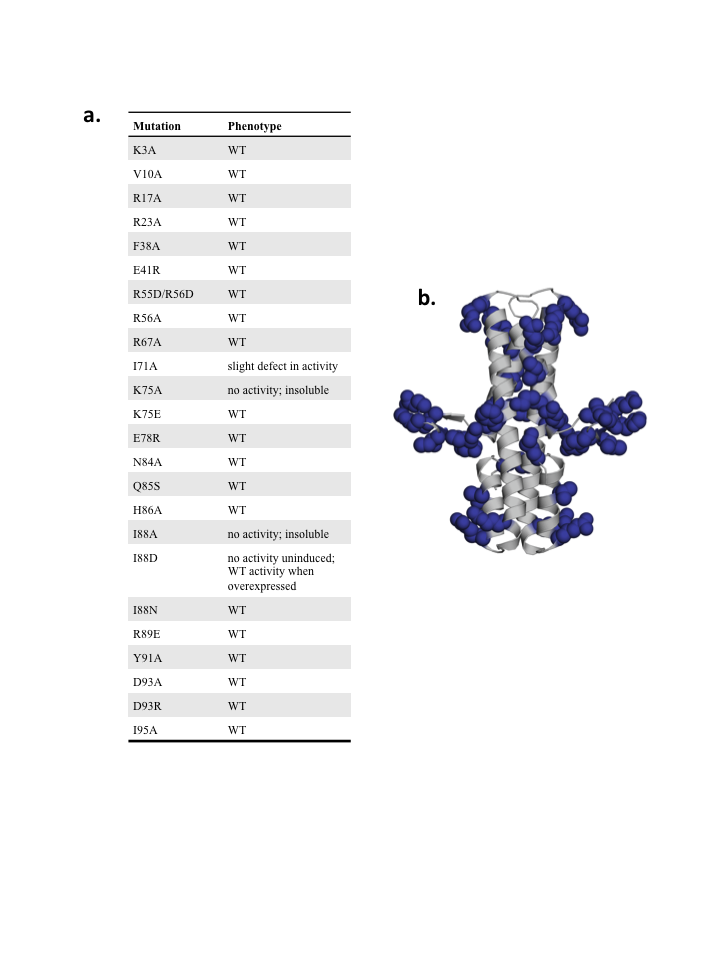

Supplement: FIG S2 [file mbo006173630sf2.tif]

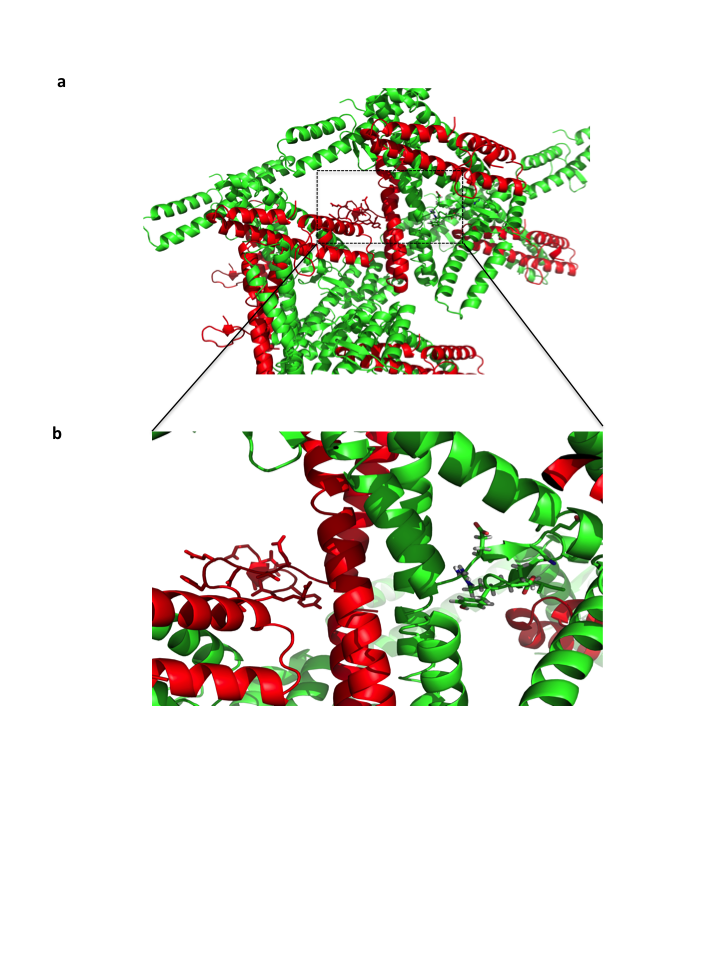

Supplement: FIG S3 [file mbo006173630sf3.tif]
