# Supplementary material for: Disabling a Type I-E CRISPR-Cas Nuclease with a Bacteriophage-Encoded Anti-CRISPR Protein
Source: mBio. 2017 Dec 12;8(6):e01751-17. doi: 10.1128/mBio.01751-17 (PMC5727412; doi:10.1128/mBio.01751-17)
Supplement: TABLE S1 [file mbo006173630st1.docx]

**Table S1**

LC-MS/MS results from the prominent ~97 kDa band excised from an SDS-PAGE gel after affinity purification of AcrE1 NHis from *P. aeruginosa* SMC4386 cells. The peptides identified were mapped to the *P. aeruginosa* PA2192 proteome. The table lists the top ten hits sorted by percent coverage.

| Protein | Accession | Size (kDa) | Unique peptide count | Coverage |
| --- | --- | --- | --- | --- |
| Cas3 | PA2G_00229 | 99 | 55 | 67% |
| Lon protease | PA2G_05917 | 89 | 40 | 52% |
| AcrE1 |  | 12 | 5 | 46% |
| Topoisomerase IV | PA2G_04988 | 83 | 9 | 14% |
| Lon protease | PA2G_00838 | 89 | 9 | 12% |
| Hypothetical | PA2G_03087 | 88 | 8 | 12% |
| Polyphosphate kinase | PA2G_04705 | 83 | 7 | 12% |
| PEP synthase | PA2G_00752 | 86 | 8 | 12% |
| DnaK | PA2G_05191 | 68 | 4 | 8% |
| GroEL | PA2G_05676 | 57 | 3 | 8% |
