# Supplementary material for: Disabling a Type I-E CRISPR-Cas Nuclease with a Bacteriophage-Encoded Anti-CRISPR Protein
Source: mBio. 2017 Dec 12;8(6):e01751-17. doi: 10.1128/mBio.01751-17 (PMC5727412; doi:10.1128/mBio.01751-17)
Supplement: TABLE S2 [file mbo006173630st2.docx]

**Table S2**

Normalized total spectral counts for elution fractions from native nickel affinity purification of formaldehyde crosslinked samples. The 6xHis-tagged anti-CRISPR protein expressed in *P. aeruginosa* SMC4386 cells for each sample is indicated at the top. Crosslinks were reversed using heat prior to LC-MS/MS analysis, and peptides were mapped to the proteome of *P. aeruginosa* strain PA2192. This table contains a selected list of hits with high numbers of peptides and high confidence of protein identification. Abundant proteins and common contaminants are detected similarly across the three samples, but only full-length AcrE1 co-purifies with Cas3.

| Protein | Accession | AcrE1^PA21^ NHis | AcrE1_Δ91-100_ NHis | AcrE3 NHis |
| --- | --- | --- | --- | --- |
| AcrE1 | n/a | 12 | 6 | 57 |
| Cas3 | PA2G_00229 | 37 | 0 | 0 |
| Ketol-acid reductoisomerase | PA2G_05259 | 155 | 65 | 68 |
| Glycerol-3-phosphate dehydrogenase | PA2G_03023 | 48 | 26 | 27 |
| Phosphoribosyl-AMP cyclohydrolase | PA2G_04886 | 10 | 6 | 10 |
| DnaK | PA2G_05191 | 2 | 7 | 10 |
| FliC | PA2G_00073 | 7 | 5 | 5 |
